# Post-piercing perichondritis

**DOI:** 10.1016/S1808-8694(15)30156-7

**Published:** 2015-10-18

**Authors:** André de Paula Fernandez, Ivan de Castro Neto, Christiane Ribeiro Anias, Patrícia Ciminelli Linhares Pinto, Jair de Carvalho e Castro, Arturo Frick Carpes

**Affiliations:** a Graduate Student; b 1st-Year Graduate Students - SCMRJ; c PhD. Graduate Program Preceptor - SCMRJ; d MsC Student. Head of the IIa Infirmary - SCMRJ; e Professor and PhD. Head of the IIa Infirmary - SCMRJ; f 1st-Year Graduate Students - SCMRJ

**Keywords:** external otitis, perichondritis, piercing

## Abstract

Piercing has become more and more popular among adolescents. The procedure is generally performed by unqualified professionals and carries its risk. Non-sterilized material or inappropiate hygiene increases the possibility of perichondritis and celulitis. The disease is characterized by erythema of the auricula pinna, unbearable pain and fever. Left untreated, the condition progresses with edema along the auricula and abscess formation that may result in ischemic necrosis and a cauliflower anesthetic deformation. The most common bacteria is Pseudomonas aeruginosa. In cases with abscesses, drainage is necessary along with antibiotic therapy guided by cultures and antibiogram. **Aim**: The aim of this case report was to review the past 10 years of published papers dealing with anatomical aspects of the auricular pinna, the history of piercing and its most common complications. **Methods**: A case report of perichondritis after “high” ear piercing that required surgical treatment and that progressed with no esthetic loss. **Results**: Theoretical and practical experience based on a review and a report of a case that progressed satisfactorily. **Conclusions**: The increased incidence of perichondritis in adolescents should require more elaborated primary prevention measures.

## INTRODUCTION

The popularity of body and ear piercings is increasing among teenagers^3^, especially when we consider the piercing of the posterior third of the ear pinna cartilage, also known as “high ear piercing”. The complications associated with piercing carried out by unqualified and untrained professionals may cause ear perichondritis. Once established and when associated with subperichondral abscess and cartilage loss, it becomes difficult to treat, causing possible cosmetic deformities, one of which is known as “cauliflower ear”, with little likelihood of a successful plastic reconstruction^2^.

In the past, most complications associated with ear piercing did not result in significant comorbidities, because most sites of implantation were in the ear lobe. At this site, infections evolve in a benign fashion, responding to local measures and anti-streptococcus antibiotics, contrary to what we have seen lately^3^. “High ear piercing” increases infection morbidity, especially because of Pseudomonas aeruginosa and its antibiotic resistance^2^.

Body piercing history, usually associated with tattooing, in the ear, mouth or nose, can be seen in practically all contemporary or primitive societies, from Asia to South America^6^. The main reasons for doing it vary from religious, rebellion or mysticism to initiation rituals or rites of passage from teenage years to adulthood.

Looking at it in broader terms, body piercing means the penetration of an object or a piece of jewelry in a previously pierced body area such as the eyebrow, ear helix, lips, tongue, nose, navel, nipples and genitals. Ear lobes and cartilages are the most commonly pierced places. Usually, the procedure is carried out without local hygiene or anesthesia, with the passage of a needle through the region and later insertion of an object into the cavity.

The piercing material varies between titanium and steel, avoiding nickel or tin - highly allergenic. Healing time varies according to the insertion site, and it can be up to one year (navel)^6^.

## LITERATURE REVIEW

Complications in the piercing site, especially in regions with low blood supply, such as the ear cartilage as in this case, may occur in up to 35% of the cases^6^.

The ear pinna is formed by a cartilaginous framework, covered by subcutaneous tissue and skin. Anteriorly, the skin is firmly adhered to the cartilage, with very little subcutaneous tissue. The latter enlarges as one moves downwards, towards the ear lobe. Posteriorly, there is more subcutaneous tissue, reducing the adherence between skin and cartilage. Cartilage nutrition is carried out by the contiguous perichondrium, and it should be preserved adhered to the cartilage in order to avoid necrosis.

The major pinna cartilage references are the helix, anti-helix, tragus, anti-tragus, scaphoid fossa, triangular fossa, ear concha and lobe, which together make up a cone set, allowing for a better sound capture, funneling it towards the external ear canal and the tympanic membrane.

Posterior (medial) Pinna”s blood supply is carried out through the posterior auricular artery (retro-auricular) which, through its perforating branches also nourishes part of the anterior helix, concha and lobe - making up an important factor in retro-auricular surgical approaches. Blood supply to the anterior (lateral) region happens, mostly, through the auricular branch of the superficial temporal artery and, in a lesser degree, through the same posterior auricular artery. Lymphatic drainage, however, follows different routes, and the lymphatic vessels of the superior portion of the pinna”s lateral face drain to the superficial peri-parotid lymphatics; the lymphatic vessels of the medial face superior portion (cranial) of the pinna drain to the mastoid lymph nodes and to those deep in the neck; and the remaining lymphatic vessels, including the lobe one, drain to the neck superficial lymph nodes.

The pinna is innervated by the greater auricular and minor occipital nerves, which branch off the neck plexus, by the auriculotemporal branch of the trigeminal nerve and the auricular branch of the vagus nerve. It is possible to achieve regional pinna anesthetic block by injecting the anesthesia in the auricular-skull sulcus.

The pinna is fixed to the skull by means of a strong insertion of the meatus cartilage to the tympanic bone and by some fragile intrinsic striated muscles innervated by the posterior auricular nerve^9^.

Perichondritis or perichondral inflammation is a severe and recently very frequent complication. The characteristic sign is ear pinna redness, except for the ear lobe (does not have cartilage). Pain, usually intense, may co-exist with fever. If treatment is delayed for unawareness or carelessness, there may be widespread pinna edema and infection spread becoming a subperichondral abscess with possible cartilage ischemic necrosis. When abscess ensues with a fluctuation aspect, there is the need for surgical drainage with necrosed tissue debridement and broad spectrum intravenous antibiotic treatment (third generation cefalosporins, fluoroquinolones and nitroimidazole) and antibiogram culture of the exudate harvested. Second intention healing process often causes an ear deformity known as “cauliflower” ear1. The destruction of ear cartilage in cases of unfavorable development, associated with a creased and deforming scar, hampers plastic reconstruction success^5^.

The most commonly found pathogen is Pseudomonas aeruginosa, together with Staphylococcus aureus^6^. Other complications are described, such as toxic shock syndrome, hepatitis, brain tetany, sarcoidosis granuloma, cystic formation, double-tail ear lobe, hematoma and deformation cheloid formation, such as systemic collateral effects such as diarrhea, headache, dysphagia, odynophagia, vomits, pyrexia and confusion.

## CASE PRESENTATION

**Figure 1:**
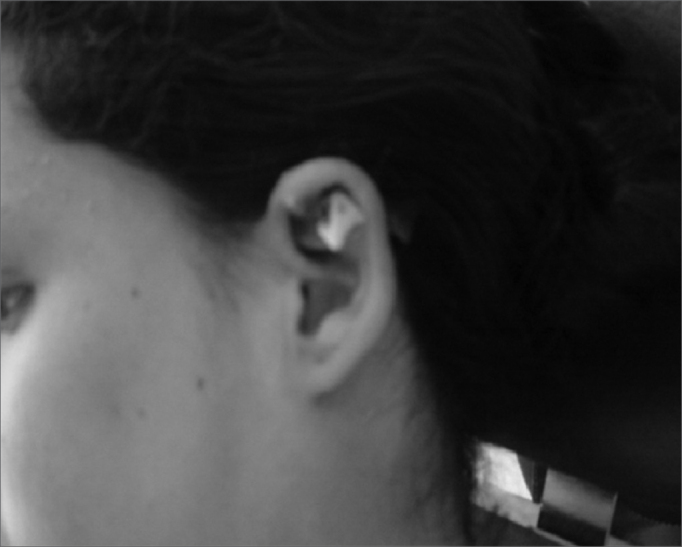
Preoperative

B.J.O., 14-year-old female Caucasian patient, comes to us in March of 2004, complaining of pain in her left pinna for one week. She had been taking monohydrated cefadroxil, 500mg bid for five days then, without clinical improvement. She had had a piercing implanted in the upper third of her left ear pinna three weeks before. Her initial physical exam showed edema, hyperemia and antero-inferior bulging of her left pinna and two regions of fluid collection that meant abscess formation, one in the upper third of the helix and another in the anti-helix region (Fig. 1). She also had ipsilateral neck lymph node enlargement. Her external acoustic meatus and tympanic membrane were normal. She had no meningism or neurological focal signs.

**Figure 2:**
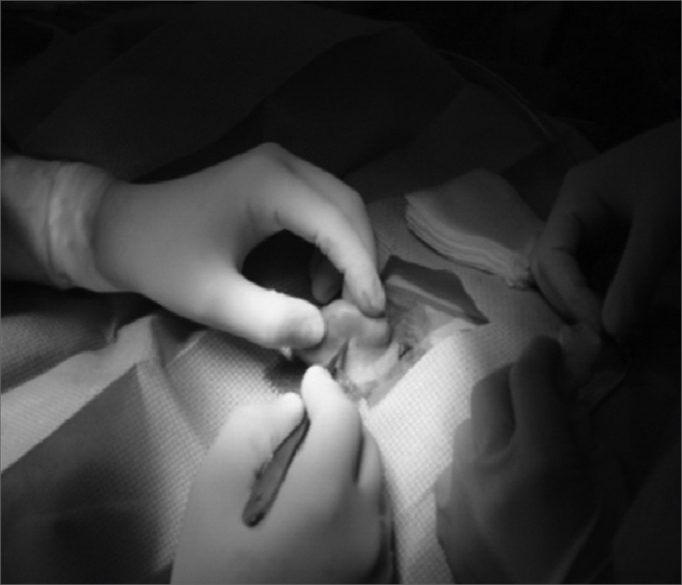
Intraoperative

The patient was admitted to the hospital and started on 500mg of aztreonam and 1g of oxacillin every 6 hours, and promptly submitted to abscess drainage under local anesthesia. During the procedure we collected the exudate for bacteriology test and we inserted two tubes to drain the fluid collection (Fig 2).

**Figure 3:**
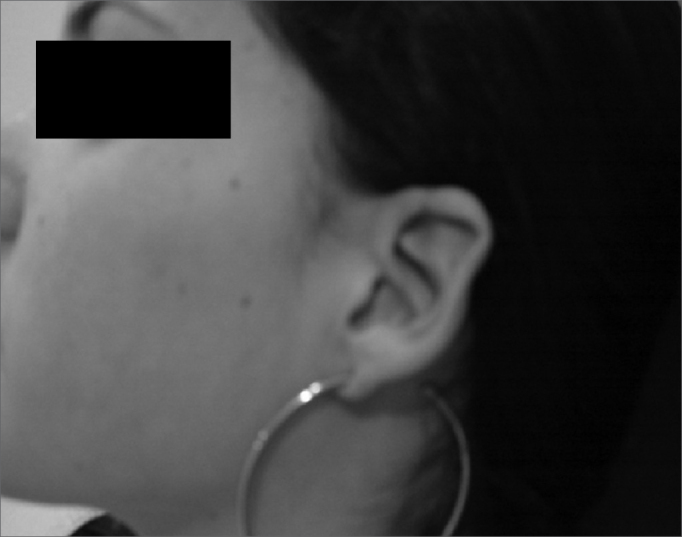
Postoperative

In the first day of post-op the patient already reported pain improvement. Her exam showed a gradual reduction on the local edema and hyperemia. Hospital discharge happened on the third day of post-op, when she was prescribed 500mg of ciprofloxacin chloridrate tid. Her exudate culture showed the growth of Pseudomonas aeruginosa. The draining tubes were removed on the fourth post-op day (Fig. 3).

**Figure 4:**
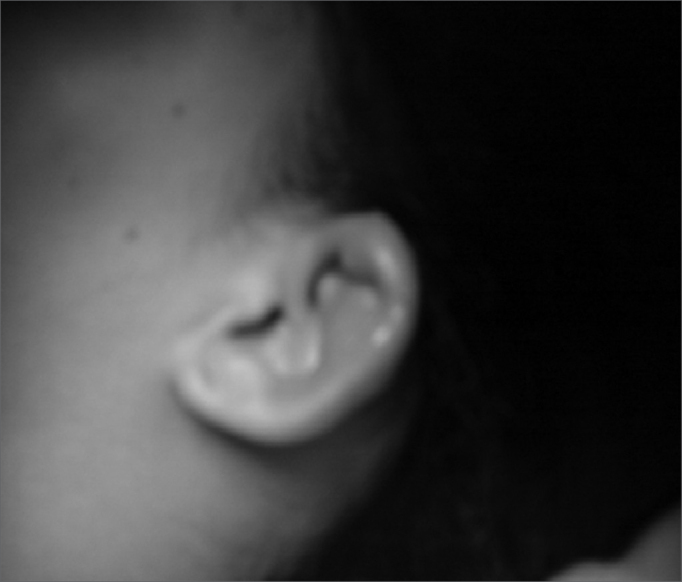
Sequelae

During the weekly follow up she showed a major improvement in the lesion aspect. She was discharged from treatment at the end of the third week of antibiotic use, without noticeable anatomic sequelae (Figure 4).

## DISCUSSION

Sexual behavior, sadism, cosmetics, mysticism or pure rebelliousness are some of the reasons given by people who have piercing implants in their bodies. It is an established fashion, with a certain appraisal in the major means of culture and advertisement (television and Internet), influencing the most volatile portion of the population - the teenagers.

Ears have been pierced for hundreds of ears now and the literature have always reported ear lobe complications caused by Staphylococcus aureus infections. The first case of pseudomonas causing pericondritis was described about 10 years ago, which reflects this new trend among young people^3^.

There are no exact statistics as to the percentage of complications, varying from 10 to 30% in many studies. However, one thing is certain: potential complications associated with piercing can be severe and must be kept in mind, especially those associated with aesthetics^2^, the spread of sexually transmitted diseases (hepatitis and HIV) and bacterial and viral infections such as tetanus, leprosy and tuberculosis, and also the systemic spread in immunodeficient individuals^6^.

Piercing is usually carried out by non-authorized or untrained professionals, who use implant techniques learned in videos or magazines or through inexperienced instructors for a period of time considered, at least, insufficient. They have no consensus on asepsis techniques, varying from Benzalkonium chloride, ethylic and isopropyl alcohol to iodine solution (the best product to eliminate Pseudomonas). These so called professionals are not aware of the risks brought about by inadequate procedures and the simple means available to avoid them.

Perichondritis usually sets in during the summer time, when air moisture and skin moisture fosters the proliferation of the most common causal agent^8^. Pain, erythema, edema and abscess formation with drainage points are characteristic, and usually develop along the time of 4 weeks after the ear implant. Surgical treatment is unavoidable when there is subperichondral involvement, aiming at surgical drainage with immediate debridement of necrosed tissue together with broad spectrum intravenous antibiotic treatment.

Pseudomonas strains, present in most of the exudative material cultures, are still very much sensitive to quinolones, which makes oral treatment an accessory to the surgical procedure. Ciprofloxacin is also efficient against a number of Staphylococcus aureus species; however its use must be restricted to patients above 18 years of age, due to the potential risk of it damaging the cartilage that is being formed4. The sooner the diagnosis is made1, the less aesthetic sequelae is seen, which can be limited to only a non-deforming hypotrophic scar.

In Brazil there is no specific law to regulate these implants, especially when they are performed in minors. One example of how this law could be more strict in Brazil is the case in Italy, where a patient, after the implant of a piece of metal in her tongue developed fatal hepatitis in less than 3 weeks, and that caused the justice department to allow piercing to be performed only by physicians and with full explanation of the risks associated with the procedure^7^.

In emergency situations, such as trauma, where the speed in the first aid makes a vital difference, the presence of a piercing can delay the patient”s care. As an example, in the USA they carried out a survey with 28 emergency room physicians, only 6 of them were able to skillfully remove a piercing implant, and this shows the importance of the different health care professionals who work with emergencies to understand how they can be removed, if needed^6^.

## CONCLUSION

Complication rates associated with piercing are mainly related to the implant site, the type of material used in sterilization, the hygiene and post-implant care and the very existence of a piercing professional^6^. Besides the development of new techniques and approaches (modifiable risk factors), the best treatment still is prevention, highlighting education on the risks of such procedure and instructions as to how to perform a better daily cleaning^5^.
